# Soybean Flour and Wheat Germ Proportions in Artificial Diet and Their Effect on the Growth Rates of the Tobacco Budworm, *Heliothis virescens*

**DOI:** 10.1673/031.009.5901

**Published:** 2009-07-17

**Authors:** Carlos A. Blanco, Maribel Portilla, Craig A. Abel, Henry Winters, Rosie Ford, Doug Streett

**Affiliations:** ^1^ USDA Agricultural Research Service, Southern Insect Management Research Unit and; ^2^ Biological Control of Pest Research Unit, Stoneville, Mississippi 38776, U.S.A

**Keywords:** tobacco budworm, intrinsic rate of increase, development time, fertility, longevity, diet preparation and cost

## Abstract

Soybean flour and wheat germ are the two most important protein components of wheat germ-based insect artificial diets. The effect of modifying the proportion of these two ingredients in a Noctuidae-specific diet was investigated utilizing the tobacco budworm *Heliothis virescens* (F.) (Lepidoptera: Noctuidae), with the goal of developing a suboptimal diet that, without drastically affecting this insect's growth and reproductive rates, could manifest subtle negative effects in this insect. The original diet formula contained 2.51% protein. When the proportions of soybean flour and wheat germ were changed to 2.15% protein the net reproductive rate of the first generation was significantly lower. In the second generation, the net reproductive rate, development time, percent female survivorship, fertility, intrinsic rate of increase, finite rate of increase and female longevity were significantly lower in both the 2.15% and 2.26% protein diets. The survival rate of immatures to the adult stage was 1% in the 2.05% protein diet in the first generation. Interestingly, females exposed to these suboptimal diets produced a significantly higher number of eggs but the survival of their larvae was significantly reduced. It is evident from these results that modifications to the protein content and the nutrient composition profile of the original wheat germ-based insect artificial formula can be used to produce subtle negative effects on the growth of tobacco budworm.

## Introduction

Insect artificial diets are essential tools for arthropod research. There are a wide range of commercial diets as well as published formulations. These diets have been developed to maximize insect growth and reproduction, by meeting or surpassing their minimum nutritional requirements. Therefore, these diets may mask potential developmental negative effects when they are used for reproductive studies that would be apparent only when the insects are exposed to sub-optimal diets. For example, insects reared on plant tissue might not develop as well as when they are reared on artificial diet because the plants' secondary chemistry and or lower nutritional value might arrest their optimal development (Blanco et al. 2008).

Soybean-flour/wheat-germ diets are commonly used to rear Noctuidae (Brewer and Martin 1976; Brewer and Tidwell 1978; Brewer and King 1979; Brewer 1981). The Shaver and Raulston (1971) formulation, an economical medium to produce large quantities of Lepidoptera, has been the basis for rearing multiple noctuid species in the USDA Agricultural Research Service facility in Stoneville, Mississippi for over 30 years. Over time, its original formula has been modified to meet different needs and to optimize costs.

Protein is an essential ingredient of the wheat germ-based diets and a component that can be easily manipulated without having considerable extra effort or expense because soybean flour and wheat germ ingredients amount to only ≈ 10% of the diet's cost. Soybean flour and wheat germ are commercially available in large quantities that, under proper storage, can produce consistent batches of diet through time. The appropriate protein content for rearing the tobacco budworm is ≈ 3% (Guerra and Bhuiya 1977). NutriSoy® flour is the main source of protein in this diet's formula as compared to wheat germ. By adjusting the proportion of NutriSoy and wheat germ the total protein of the diet can be reduced and this manipulation might result in small negative developmental and reproductive effects on *Heliothis virescens* (F.) (Lepidoptera:Noctuidae). In this way, a suboptimal diet could be developed that might stress the insect's development as compared to a diet composed entirely of plant tissue, that could allow the identification of physiological and reproductive effects caused by suboptimal nutrition for ecological and reproductive biology studies.

This study had the goal of lowering the protein content of the Shaver and Raulston (1971) formula, to decrease the reproductive potential of the tobacco budworm, such that artificial diet might more accurately reflect this insect's development on its plant hosts. Our hypothesis was that reduction of protein content would result in a decline in biological fitness of this species thus unmasking the effects of “over-nutrition” and allowing nonnutritionally-based fitness problems to be expressed.

## Materials and Methods

This study was conducted using the *H. virescens* colony of the USDA Agricultural Research Service of Stoneville, Mississippi, a colony that was initiated in 1971 from larval collection from wild host plants. Neonates (300 ≤ 16 hold) from the same cohort (P_0_ generation) were placed individually in plastic cups (37-ml [T-125 Solo® plastic soufflés, www.solocup.com]) containing ≈15 ml of one of the diets described in Table 1. These diets reduced the proportion of protein by reducing the amount of NutriSoy® (which contains 40–42% protein) and increasing the amount of wheat germ (which contains 29% protein). Cups were closed with cardboard lids and kept in an incubator at 27 (± 0.4) °C, 75 ± 10 % RH and a 14:10 L:D photoperiod. Larval developmental time, immature mortality (eggs, larvae, pupae), and adult eclosion and sex were recorded for the parental (P_0_), F_1_ and F_2_ generations on a daily basis.

A sample of recently emerged (≤ 72 h) F_1_ and F_2_ moths was used to set-up 18 pairs (replicates) from each treatment, except treatment 5 that had only 3 moths on the P_0_ generation. Individual pairs were held in 500 ml containers (Model 42505LY, Consolidated® Plastic Co., www.consolidatedplastics.com) with free access to 10% sucrose solution, capped with Batist cloth (Zweigart®, www.zweigart.com) and maintained in incubators as previously described. Cloths with eggs were replaced daily form each pair, the number of eggs was estimated visually and placed in sandwich bags (Ziploc® 94600, www.Ziploc.com) in incubators set as previously described for larval hatching. Egg visual estimation was previously determined based on the comparison of the following three eggs counts: a) experienced personnel made visual estimations of eggs on cloth, b) eggs were counted on 20% of the area of the same egg cloth samples under magnification with a microscope and counts were extrapolated to total area (65.03 cm^2^ ), and c) eggs on 20% of the area of the same egg cloth samples were counted using the software Image Pro-Plus for Windows and extrapolating to total area. The three different egg-counting methods did not differ statistically (*F* = 0.06, *P* = 0.94, df = 2, 24). Using the personnel's visual estimation departed 20.04 ± SEM 4.1% from the absolute counted area mean.

A set of 300 ≤ 16-h old neonates obtained simultaneously from the 18 pairs from the P_0_ and F_1_ generations was used to set-up the next generation for each treatment. Moth pairs were maintained in their containers until death to assess longevity. The position of the moth containers inside the incubators was arranged daily in a different pattern.

**Table 1.**
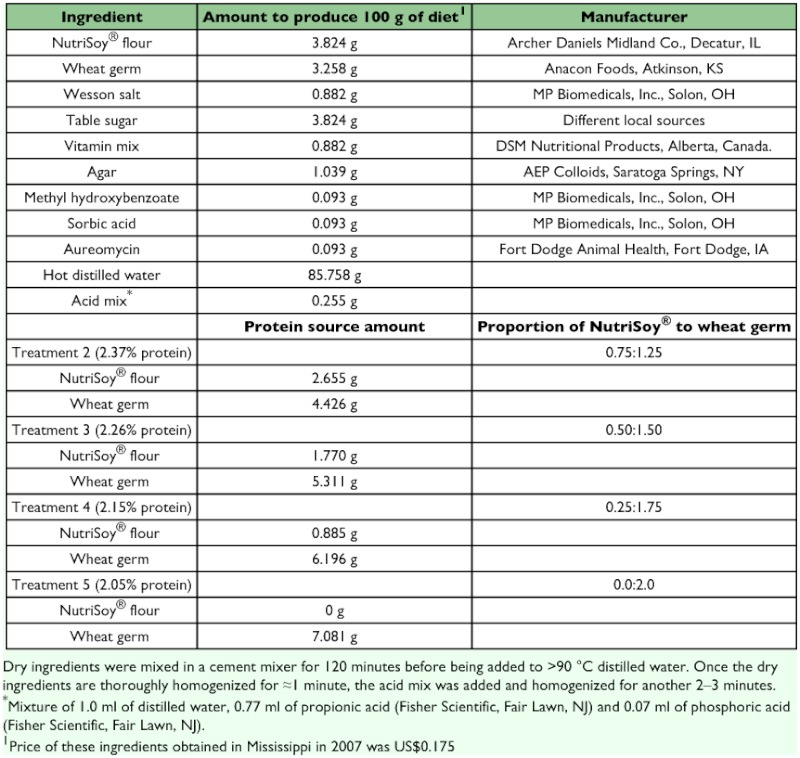
List of ingredients and amounts of the modified Shaver and Raulston's diet.

## Demographic Rates of Increase

The net reproductive rate *R*_0_, represents the mean number of female offspring produced by each female during its entire lifetime (Carey 1993). It was calculated as:

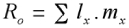

The generation time (*T*), which is the mean interval separating the birth of one generation from the birth of the next, was calculated as:

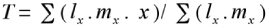

Where survival (*l_x_*) is the probability of an individual attaining age *x*, and net fertility (*m_x_*) is the mean number of individual females produced per female of age *x*. The *m_x_* value was determined by multiplying the mean number of eggs produced per female at age *x* by their sex ratio.

The intrinsic rate of increase (*r_m_*), is a special case of a crude growth rate, where its exact *r* value was determined from survival and reproduction by substituting trial values of *r* based on life fecundity tables constructed using Lotka's equation (Carey 1993; Krebs 2001).

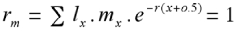


The intrinsic rate of increase was then used to calculate the finite rate of increase (**λ**), denoted as the population increment (female and male individuals/female/generation based on *r* value), and the doubling time (*dt*), which is the expression for geometrical increase.

## Statistical Analysis

The net reproductive rate, developmental time, doubling time, gross and net fecundity were calculated by constructing individual life tables for each pair (1 ♀: 1 ♂ / replicate) per treatment per generation. Therefore, for the individual pair tables *l_x_*= 1 for all values of *x*, so *R_0_* has the same value as mean age of gross fecundity (*M_x_*) (mean number of individual females and males produced per female of age *x*) (Baker et al. 1992). To obtain more accurate results the values of the 300-unit sample size used to calculate the mortality of immature stages and sex ratio were incorporated into the life tables to estimate the *l_x_* and *m_x_* parameters (Portilla et al. 2000). Data (means ± SE) of demographic parameters presented in table 2 were analyzed using a one-way analysis of variance (ANOVA) by the general lineal model (GLM) procedure of SAS (9.1). Differences between least square means for all variables for each treatment and for each generation (calculated by the GLM procedure) were evaluated by *t*-test in SAS (9.1). Regression analyses were used to determine the correlation between net reproductive rate (*R_0_*) and soybean flour/wheat germ proportions (treatments).

**Table 2.**
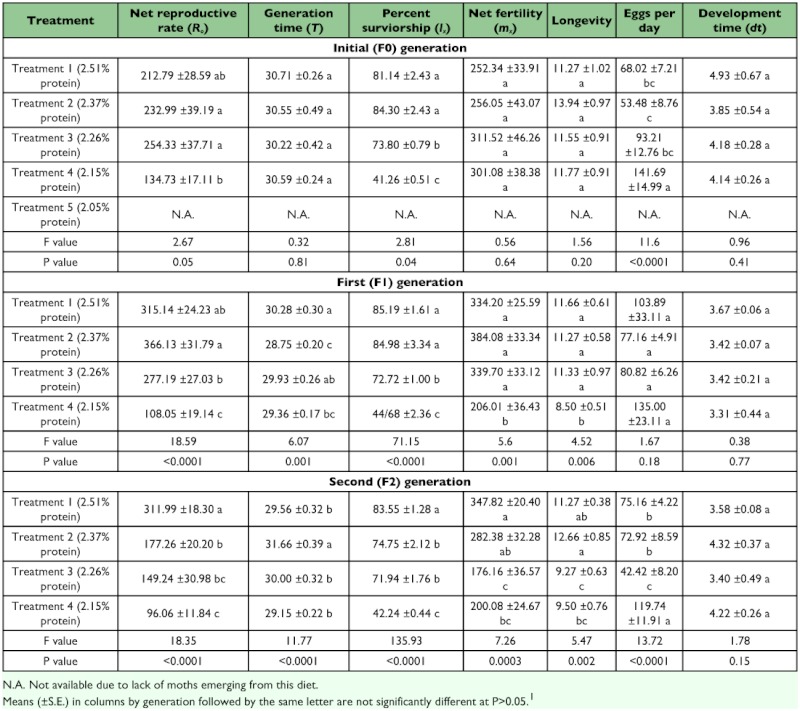
Effect of different diet's protein content on the development of three sequential generations of *Heliothis virescens*.

## Results

Wheat germ alone (treatment 5, 2.05% protein, 0% soybean flour: 100% wheat germ content) could not maintain this tobacco budworm colony. Although a small proportion (1.3%) of larvae reached pupation, the resulting moths (1 ♀ and 2♂) did not produce progeny. The negative effect of this treatment was so drastic that its use would not result in the desired subtle effects for reproductive biology studies. Protein content manipulation might not be the only factor behind these results. The addition of extra amounts of wheat germ changed the nutritional composition profile of the diet due to the fact that wheat germ contains antinutrients and digestive enzyme inhibitors (Cohen 2004).

The net reproductive rate (*R_0_*), of larvae was negatively affected in the initial (P_0_) generation for treatments 2 (2.37% protein) and 4 (2.15% protein), and in the subsequent (F_1_) generation for treatment 3 (2.26% protein). Thus, a reduction in protein of ≥ 5.5% had a negative effect on reproduction. An interesting phenomenon was noticed with moths emerging from treatment 4. Those females laid a significantly higher number of eggs but larval survivorship (*l_x_*) was negatively affected (Table 2). In the first generation, the net reproductive rate (*R_0_*), development time, percent immature survivorship, net fertility (*mx*), intrinsic rate of increase (*rm*), finite rate of increase (λ) and female longevity were also significantly reduced with treatment 4, and some of these effects were also noticeable in treatment 3 as well (Tables 2 and 3). The same reproductive and developmental parameters were significantly affected in the second (F_2_) generation, with the exception of development time, further corroborating the effect of lower protein levels on development of *H. virescens*.

**Figure 1.**
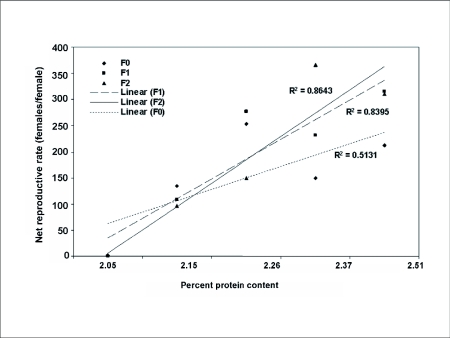
Correlation between Net reproductive Rate (R_0_) of *Heliothis virescens* and the percent of protein content in insect artificial diets.

In the second generation, the effects on some of the above mentioned parameters began to be apparent in *H. virescens* fed treatment 2 (2.37% protein) (Table 2 and 3). Net reproductive rate, net fertility and longevity were negatively affected, while eggs per day significantly increased for treatment 3 (2.26% protein) following the same pattern previously found on the first generation with treatment 4 (2.15% protein). Regression analysis performed with net reproductive rate (*R_0_*) values made these trends also noticeable (Figure 1). The first generation was not greatly affected by the treatments (R^2^ = 0.51), but the subsequent generations due to the effect of suboptimal treatments (≤ 2.26% protein), produced a clear effect on net reproductive rate (R^2^ = 0.83 for F_1_ and R^2^ = 0.86 for F_2_). Similar tendencies were also noticed with two other important parameters, cumulative gross fecundity and immature survival that were negatively affected by some of the treatments when *H. virescens* were exposed for more than two generations to a suboptimal diet.

**Table 3.**
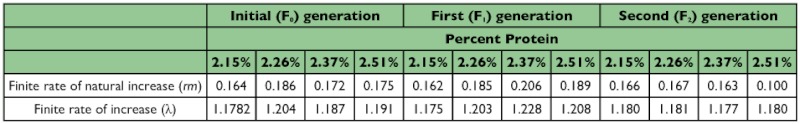
Effect of protein content on finite rate of natural increase and finite rate of increase of three sequential generations of *Heliothis virescens*.

Figure 2 shows similar cumulative gross fecundity patterns for all treatments and generations where egg production rapidly reached its peak by day 11. The highest cumulative gross fecundity value was recorded for treatment 1 (2.51% protein) in the initial generation (654.62 individuals/female). Immature survival decreased considerably on larvae fed treatment 5 (2.05% protein) after the first day and became more pronounced after 14 days, whereas in treatment 4 for all generations the decrease was delayed until after the 25^th^ day. The highest immature survival was obtained on individuals fed treatment 1 (all generations) and treatment 2 (initial and first generation) (Figure 3).

## Discussion

Subtle differences between diet components can have an effect on noctuids' feeding preferences (Moore 1986) or on their development. An insect artificial diet that can produce suboptimal growth may reflect more accurately the development pattern of noctuids been fed certain plant tissue. Because most of the noctuids plant hosts contain some type of ‘noxious phytochemicals’ capable of interfering with proper nutrient assimilation on insects (Whittaker and Feeny 1971), a suboptimal insect artificial diet may simulate insect growth on plant tissue. Additions of naturally-occurring plant components such as gossypol (Gunasena et al. 1988) or nicotine (Gunasena et al. 1990) to insect artificial diet can also achieve lower developmental rates on certain insects as well, such as it is the case of the tobacco budworm. However, adding these plant components can be expensive or the specific chemicals might be difficult to obtain. Since wheat germ is an essential ingredient in many diets providing ≈30% less protein than soybean flour (Cohen 2004), an elevated proportion of this component, that provides ≥ 68% of the total protein content of the diet, can produce easilymeasured negative effects on the development of tobacco budworm such as percent survivorship (*lx*). Other easyto-document parameters such as longevity and egg production were not constant in all generations or appeared only after one generation (longevity), but are important quantifiable parameters that their differences can be noticeable with these suboptimal diets.

Manipulations of the diet's protein ingredients as described in this study might be one way to notice developmental and reproductive parameters that otherwise cannot be noticeable when insects are fed with diets that surpass all the minimum developmental requirements. One of the advantages of producing a suboptimal insect artificial diet that can simulate some of the effects found on *H. virescens* when fed on plant tissue is that an artificial diet may be more homogenous in its nutritional contents compared with the potential nutritional and/or chemical variation that the environment can produce on fieldgrown plants. The development of *H. virescens* on 2.05% protein closely simulated its development on garbanzo beans (*Cicer arietinum*) and white clover (*Trifolium repens*) (Blanco et al. 2008).

The interpretation of these results could be mitigated by the fact that the *H. virescens* colony used has been strongly selected to feed on the Sharver and Raulston (1971) diet for hundreds of generations.
